# Potential Neurobiological and clinical markers in Extreme Weight Conditions: from Anorexia to Obesity

**DOI:** 10.1192/j.eurpsy.2024.35

**Published:** 2024-08-27

**Authors:** F. Fernandez-Aranda, S. Jimenez- Murcia, Psychoneurobiology of Eating and Addictive Behaviours Group, Neuroscience Program, IDIBELL

**Affiliations:** ^1^Clinical Psychology, University Hospital of Bellvitge-IDIBELL; ^2^CIBERobn, Instituto Salud Carlos III; ^3^Clinical Sciences, University of Barcelona, Barcelona, Spain

## Abstract

**Abstract:**

Extreme eating and weight conditions (EWC) are a construct that emerges as a dimensional and theoretical model that identifies individuals who exhibit inappropriate eating behaviours and abrupt weight fluctuations. According to this spectrum of EWC, one extreme can be represented by individuals with anorexia nervosa (AN), characterised by excessive food restriction and an extremely low body mass index (BMI), whereas the other end of this continuum is represented by individuals with obesity (OB), characterised by a BMI above 30. In addition to AN and OB, some eating disorders (EDs), namely bulimia nervosa and binge eating disorder, are also part of this continuum, given the high risk of falling into one of the extremes, especially that of higher BMI. Studies have described similar changes at the psychological and neurobiological levels associated with their abnormal eating patterns, delineating vulnerability pathways related to the neurobiological basis.

Based on previous literature, individuals suffering from EWC would show dysfunctional brain activity in regions associated with emotional reward processing and cognitive control compared to healthy controls (HC). Similarly, neuroendocrine alterations in EWC are expected to influence clinical symptomatology. It will also be discussed how impairments in executive function and differential brain activity observed in individuals with EWC may negatively impact their clinical course and treatment outcome.

**Disclosure of Interest:**

F. Fernandez-Aranda: None Declared, S. Jimenez- Murcia Grant / Research support from: We thank CERCA Programme/Generalitat de Catalunya for institutional support. This research was supported by grants from Instituto de Salud Carlos III (ISCIII) (FIS PI20/00132) and co-funded by FEDER funds/European Regional Development Fund (ERDF), a way to build Europe. CIBERObn is an initiative of ISCIII. Additional support was received from the Delegación del Gobierno para el Plan Nacional sobre Drogas (2021I031) and Ministerio de Ciencia e Innovación (grant PID2021-124887OB-I00). Additional funding was received by AGAUR-Generalitat de Catalunya (2021-SGR- 00824), European Union’s Horizon 2020 research and innovation program under Grant agreement no. 847879 (PRIME/H2020, Prevention and Remediation of Insulin Multimorbidity in Europe) and the European Union’s Horizon Europe research and innovation program under grant agreement No 101080219 (eprObes)., Consultant of: FFA and SJM received consultancy and speakers honoraria from Novo Nordisk.

